# Apoptosis in Leukemic Cells Induced by Anti-Proliferative Coumarin Isolated from the Stem Bark of *Fraxinus rhynchophylla*

**DOI:** 10.4014/jmb.2006.06022

**Published:** 2020-07-20

**Authors:** Beom Zoo Lee, Ik Soo Lee, Chau Ha Pham, Soon-Kyu Jeong, Sulhae Lee, KwangWon Hong, Hee Min Yoo

**Affiliations:** 1 Department of Food Science and Biotechnology, College of Life Science and Biotechnology, Dongguk University, Goyang 10326, Republic of Korea; 2 Chemland Co., Ltd., Gunpo IT Valley, Gunpo 15850, Republic of Korea; 3 Herbal Medicine Research Division, Korea Institute of Oriental Medicine, Daejeon 34054, Republic of Korea; 4 Group for Biometrology, Korea Research Institute of Standards and Science (KRISS), Daejeon 34113, Republic of Korea; 5 Department of Microbiology and Molecular Biology, Chungnam National University (CNU), Daejeon 34134, Republic of Korea

**Keywords:** *Fraxinus rhynchophylla*, coumarin, leukemia, apoptosis, ROS, cytotoxicity

## Abstract

Esculetin 6-O-β-D-arabinofuranosyl-(1 → 6)-β-D-glucopyranoside (EAG) is a coumarin glycoside isolated from the stem bark of *Fraxinus rhynchophylla*. This study scrutinized the anti-proliferative activity of EAG on blood cancer-derived Jurkat leukemic cells. Cell viability assays in leukemic cancer cells determined that EAG possesses potent anti-proliferative effects. Moreover, treatment with EAG increased the proportion of apoptotic cells, resulted in cell cycle arrest being induced at the subG0/ G1 phase, and reduced the proportion of cells present in the S phase. In addition, mitochondrial membrane potential was reduced by EAG in Jurkat cells. Additionally, EAG triggered apoptosis that was mediated by the downregulation of BCL-XL, p-IκBα, and p-p65 expressions in addition to the upregulation of cleaved Caspase 3 and BAX expressions. These findings revealed that the toxic effect of EAG was mediated by intracellular signal transduction pathways that involved a mechanism in which reactive oxygen species (ROS) were upregulated. Thus, this study concludes that EAG could potentially serve as a therapeutic agent for leukemia.

## Introduction

Leukemia is a prevalent form of cancer that is known as a major cause of cancer-related child mortalities [10, 25]. Leukemia consists of a group of diseases found in whole peripheral blood in addition to bone marrow that are caused when abnormal white blood cells undergo uncontrolled proliferation [26]. As a type of blood cancer, leukemia is biologically heterogeneous and is categorized across various subgroups [9]. The four main subgroups are Chronic Myeloid Leukemia (CML), Chronic Lymphocytic Leukemia (CLL), Acute Lymphoblastic Leukemia (ALL), and Acute Myeloid Leukemia (AML) [12, 13].

Since ancient eras, humans have developed medicinal remedies through the use of natural products. Examples include a multitude of secondary metabolites that have the potential to prevent or treat major diseases. Such secondary metabolites from natural products are continually used in drug development to provide key scaffolds, with many found to exhibit diverse biological actions, including anticancer activity [11].

*Fraxinus rhynchophylla* Hance (Oleaceae), a deciduous tree found in various locations in Korea and China, is one of the most commonly used plants in traditional Chinese medicine. The stem bark of this tree is mainly used as an anti-inflammatory, anti-bacterial, and analgesic agent. Furthermore, the stem bark has been reported to exhibit a multitude of pharmacological and medicinal activities, all of which are attributable to its anticoagulant, diuretic, and anti-allergic properties [22]. This plant contains numerous biologically active compounds, such as secoiridoid glucosides, flavonoids, lignans, coumarins, and simple phenolic compounds [14, 16, 23, 24]. Of these compounds, coumarins are a notable component of this plant.

In this study, we discovered that significant anti-proliferative activity against Jurkat leukemic cells was exhibited by a coumarin glycoside isolated from the stem bark of *F. rhynchophylla*, specifically esculetin 6-O-β-D- arabinofuranosyl-(1→6)-β-D-glucopyranoside (EAG). To clarify the anti-leukemia mechanisms of EAG, we studied how EAG induced apoptosis on Jurkat cells in vitro. In addition, we also determined that EAG reduced mitochondrial membrane potential, increased sub-G1 cell population, and induced ROS production.

## Materials and Methods

### Plant Material

Stem bark of *F. rhynchophylla* was collected in Namyangju-si, Gyeonggi-do, Korea, in 2017, and was identified by Professor Ki Hwan Bae of Chungnam National University (CNU), Republic of Korea. A specimen (KIOM-321) has been deposited in the Herbarium of the Korea Institute of Oriental Medicine (KIOM), Republic of Korea.

### Isolation of EAG

Air-dried *F. rhynchophylla* stem bark was extracted using 70% ethanol at room temperature for a week and was subsequently filtered and concentrated to yield a 70% EtOH extract. The extract was subjected to a series of chromatographic steps, resulting in the isolation of EAG. The detailed procedure has been described in a previous study [17].

### Cell Culture

Jurkat cells were purchased from the American Type Culture Collection (ATCC, USA) and cultured in Roswell Park Memorial Institute (RPMI) 1640, (Thermo Fisher Scientific, USA) supplemented with fetal bovine serum FBS, (Thermo Fisher Scientific), 10% and penicillin/streptomycin (Thermo Fisher Scientific), 1%. The cells were subcultured in a humidified 37°C incubator with 5% CO_2_ and were subcultured when the cell cultures reached 70– 80%.

### Antiproliferation Assay

The cells were cultured in 96-well tissue culture plates with a density of 5 ~ 6 × 10^4^ cells per well in RPMI 1640 for 24 h. After replacing the medium with a fresh complete medium containing various concentrations (10, 20, and 50 μM) of the compounds, the cells were incubated for 48 h. Treatment with dimethyl sulfoxide (DMSO) cells was considered as a control. The anti-proliferative activity of EAG on the cells was determined through the use of the CellTiter Cell Proliferation Assay Kit (Promega, USA). To estimate cell viability, the cells were incubated with the assay reagent at 37°C for 2 h. The OD (490 nm) was measured using a plate reader.

### Microscopy

The cells were seeded in 6-well plates up to a density of 1 × 10^6^ cells in each well and were treated with different concentrations of EAG (10, 20 μM). Following 24 h of drug treatment, by using a phase-contrast microscope (Olympus, Japan), the morphological characterization of the cells was photographed by a camera at 400× magnification.

### Apoptosis Assay

The cells were cultured in 6-well plates and exposed to each concentration of EAG (10, 20 μM). Following 24 h of drug treatment, the Annexin V/7AAD Apoptosis Detection Kit (BioLegend, USA) with propidium iodide (PI) was used to analyze the Jurkat cells treated with EAG via flow cytometry. The cells were washed with PBS (Thermo Fisher Scientific, USA) and centrifuged for 3 min. Subsequently, binding buffer (100 μl) was added and the cells were incubated with 5 μl of annexin V/7AAD for 20 min at room temperature (RT) in the dark. Before conducting the measurements, additional binding buffer (400 μl) was added. A flow cytometer was used to detect cell apoptosis.

### Cell Cycle Progression

The cells were seeded in T25 flasks with a density of 1 × 10^6^ cells/ flask. After 24 h, the final concentrations of EAG (10, 20 μM) were added to each respective flask, which were then incubated for 24 h. The cells were collected and fixed in test tubes with 80% cold EtOH, which was followed by incubation at 4°C for 1 h. Following the centrifugation of the cells, the cell pellets were resuspended using the reagent, which included propidium iodide (intercalating DNA dye) and RNAse A from the Muse Cell Cycle Assay Kit (Luminex Corporation, USA). The incubation of the cells lasted 30 min. The calculation of cell cycle distribution involved the use of a flow cytometer, and the FlowJo software was used to analyze the percentages of cells in the G0/G1, S, and G2/M phases.

### Mitochondrial Membrane Potential (MMP) Assay

Jurkat cells treated with EAG were harvested after 24 h, washed, and incubated with the 100 nM of tetramethylrhodamine methyl ester perchlorate (TMRM, Thermo Fisher Scientific, USA) for 30 min at 37°C. To measure mitochondrial membrane potential (MMP), the cells were resuspended and were analyzed using a flow cytometer and the FlowJo software.

### Reactive Oxygen Species (ROS) Assays

To measure ROS levels, we used 2′,7′-dichlorodihydrofluorescein diacetate acetyl ester (DCFDA). The cells were resuspended with PBS buffer at 37°C and photographed using a fluorescent microscope. Then, 1 μM of DCFDA was added to the cells and the mixture was incubated at room temperature for 30 min, washed, and resuspended in FACS buffer. Fluorescence was analyzed using a flow cytometer (BD Biosciences, USA) and the FlowJo software.

### Protein Extraction and Western Blot

The Jurkat cells were cultured at 2 × 10^5^ cell/ml in T-25 flasks and treated with EAG (10-20 μM) for 48 h. The harvested cells were lysed with RIPA buffer containing phosphatase inhibitor cocktails and protease. Protein content was quantified using bicinchoninic acid (BCA) assay reagents (Bio-Rad, USA). An equal amount of protein was separated using Bis-Tris Gel 4-12%, which was transferred onto a Polyvinylidene difluoride membrane then western blotted with the specific antibodies for cleaved-Caspase 3, BAX, BCL-XL, phopho-IκBα, IκBα, phopho-p65, p65, and β-Actin at 4oC overnight with gentle shaking. Bound antibodies were detected using Immobilon Chemiluminescent. Images were taken with an ImageQuant LAS 4000 mini (Multi Gauge, Japan).

### Statistical Analysis

GraphPad Prism was used for statistical analysis and the data were presented as means ± SEM. The results were also analyzed through one-way ANOVA and *t*-tests. *p* values less than 0.05 were regarded as statistically significant.

## Results

### Chemical Structure of EAG

**Fig. 1:**
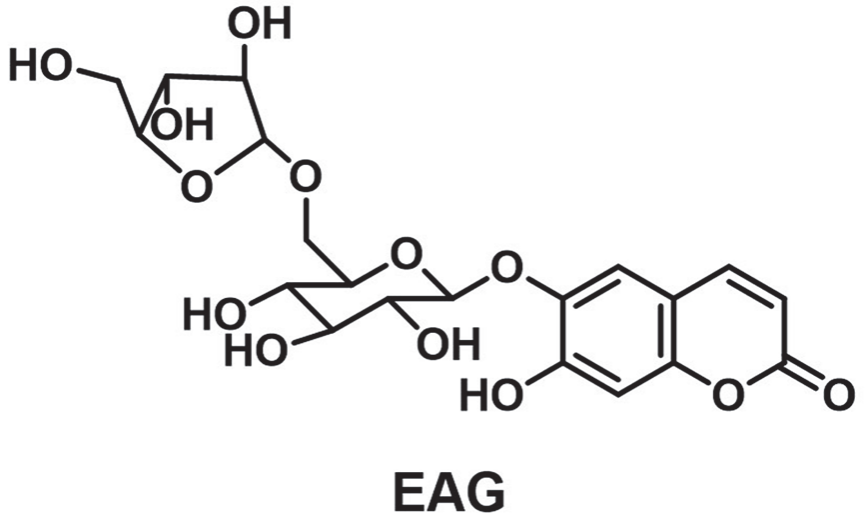
Chemical structure of EAG isolated from *F. rhynchophylla*.

Column chromatography was performed on an EtOH extract of *F. rhynchophylla* stem bark. Based on thin-layer chromatography results, the extract was separated into four fractions (A–D). Fraction C was subjected to chromatographic separation steps, leading to the isolation of a coumarin glycoside, which was determined as EAG through chemical evidence and studies based on spectroscopy (Fig. 1) [17].

### EAG-Induced Antiproliferative Effects

**Fig. 2:**
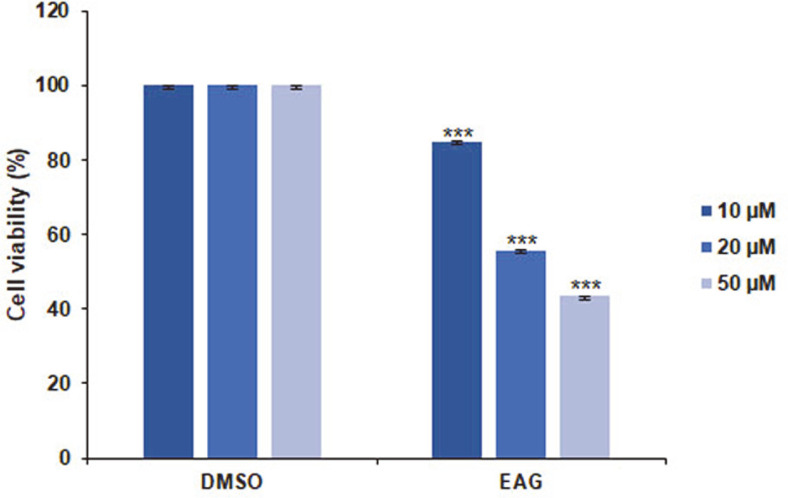
Anti-proliferative effects of EAG on Jurkat cells. Cell viability measurements using the MTS assay at 48 h post treatment. Values are represented as means ± SEM. (*n* = 3, ***p ≤ 0.001).

**Fig. 3:**
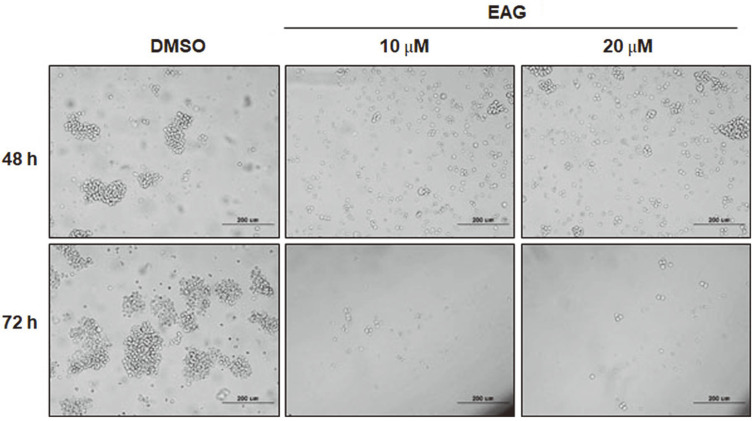
Morphology of Jurkat cells treated with EAG. Cells were observed through phase contrast microscopy (400× magnification). Cells treated with 10 or 20 μM EAG exhibit morphological differences that are features of apoptosis at 48 and 72 h post treatment.

The anti-proliferative effects of EAG on a blood cancer-type Jurkat cell line were assessed in a cell proliferation assay using CellTiter. As a result, EAG was found to exhibit dose-dependent anti-proliferative activity against Jurkat cells (Fig. 2). Treatment with EAG (20 μM) dramatically reduced cell viability, and images from a microscope showed that EAG induced changes in morphology compared to the DMSO-treated cells that were used as the control (Fig. 3). These results suggest that EAG possesses strong cytotoxic effects against Jurkat cells.

### EAG-Induced Apoptosis in Leukemic Cells

**Fig. 4:**
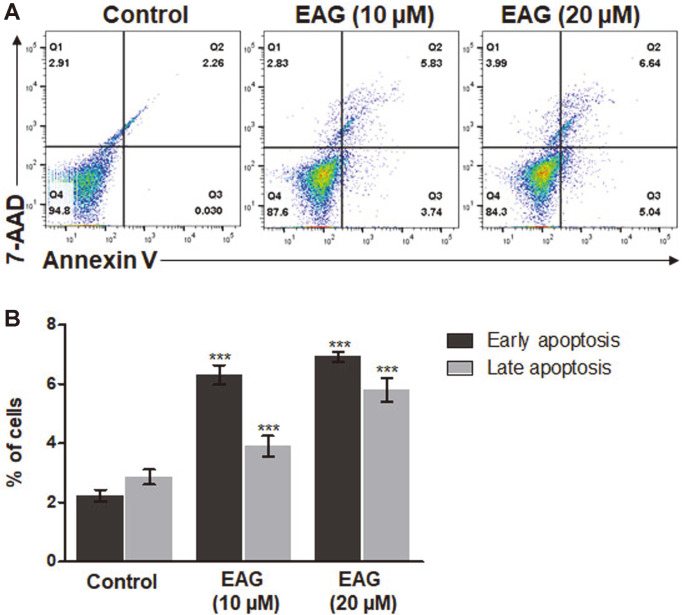
EAG-induced apoptosis in Jurkat cells. (**A**) Apoptosis analyzed using Annexin V-PE/7AAD double staining and flow cytometry. Jurkat cells were either treated or untreated with EAG for 48 h (**B**) Quantification of early and late apoptotic cells. Values are represented as means ± SEM. (*n* = 3, ***p ≤ 0.001).

The apoptosis assay showed that EAG caused Jurkat cells to undergo early and late stages of apoptosis. In the untreated cells, there were low numbers of early and late apoptotic and necrotic cells, as indicated by the minimal cell distribution in Q4. However, EAG treatment resulted in a varied cell distribution. As the concentration dose increased, there was a gradual increase in the percentage of early and late apoptotic cells, with values of 2.29%, 9.57%, and 11.68% following 24 h of incubation (Fig. 4A). The results indicate that apoptosis was augmented by EAG in leukemia cells (Fig. 4B).

### EAG-Induced Cell Cycle Arrest in Leukemic Cells

**Fig. 5:**
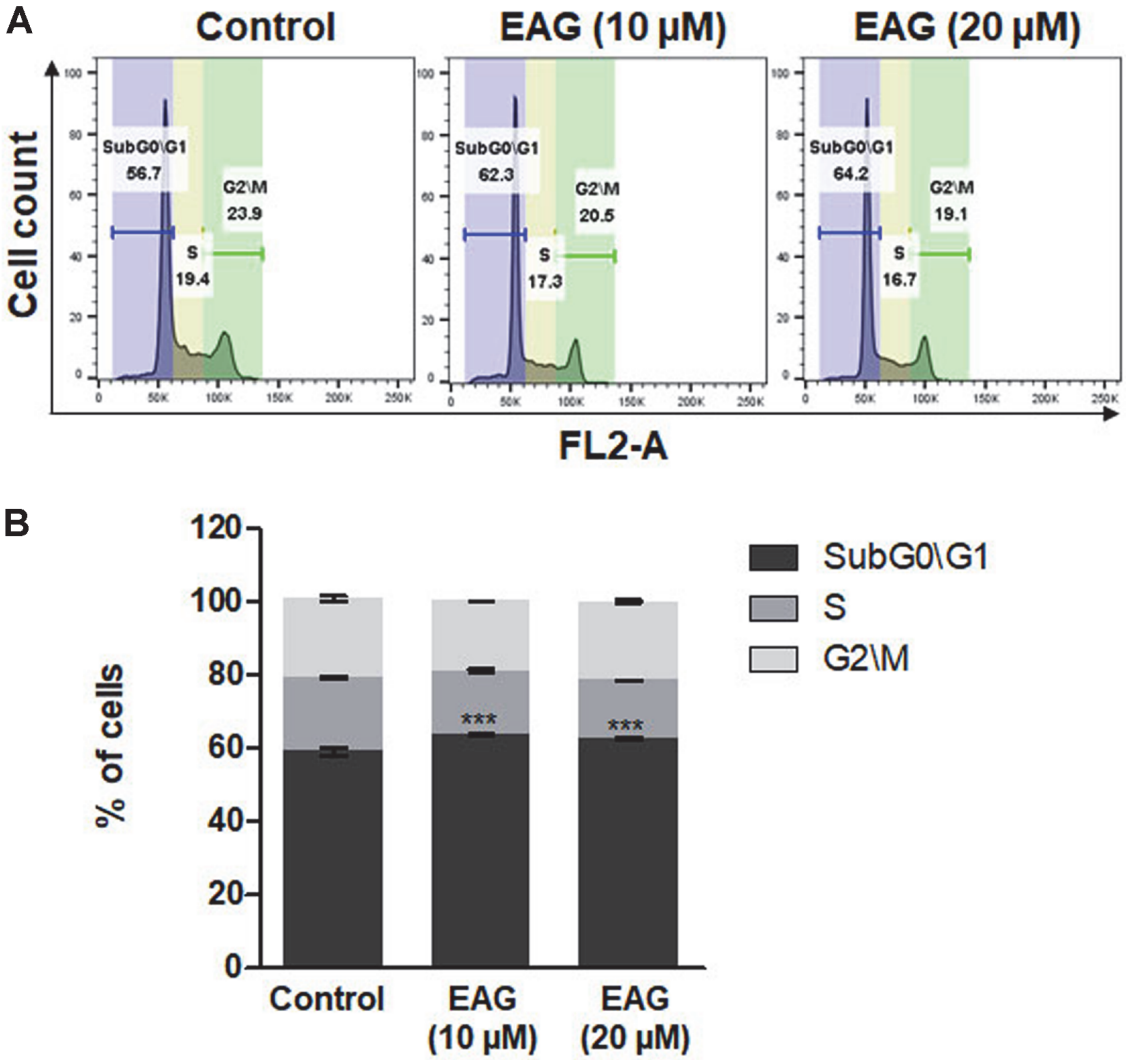
EAG-induced cell cycle differences in Jurkat cells. (**A**) Cell cycle analysis of cells treated with EAG. The cells were stained with a propidium iodide (PI) reagent for 30 min and analyzed using flow cytometry. (**B**) Analyzed cell percentages in SubG0/G1, S, and G2/M phases. Values are represented as means ± SEM. (*n* = 3, ***p ≤ 0.001).

Cancer represents uncontrolled cell division [2]. To evaluate whether cell cycle arrest was the cause that induced cell cytotoxicity, this study analyzed cell-cycle progression via flow cytometry. As Jurkat cells were treated with EAG, the sub-G0/G1 phase saw a cell percentage increase, whereas the S phase saw a decrease in cell percentage (Figs. 5A and 5B). Previous reports support the hypothesis that Sub-G0/G1 cell deaths can be caused by apoptosis [29]. Our results demonstrated that EAG inhibited the proliferation and viability of Jurkat leukemic cells by inducing cell cycle arrest.

### Effects of EAG on Mitochondrial Membrane Potential in Apoptotic Cells

**Fig. 6:**
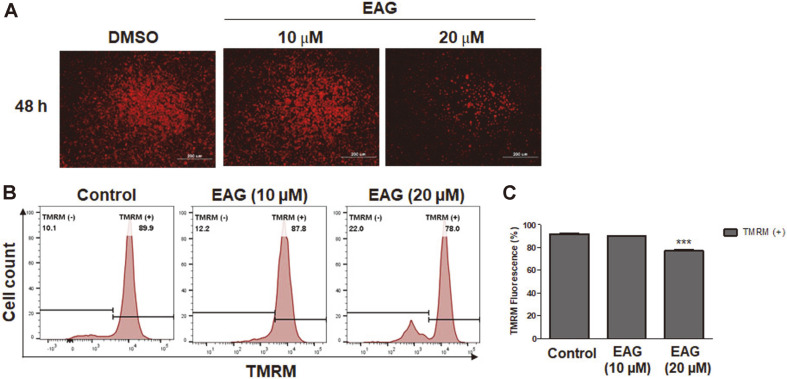
Reduction of the mitochondrial membrane potential (MMP) in Jurkat cells. (**A**) TMRM detection by fluorescence microscopy. (**B**) Mitochondrial membrane potential (MMP) of Jurkat cells incubated with TMRM, detected via flow cytometry. (**C**) Quantification of the mitochondrial membrane potential. Values are represented as means ± SEM. (*n* = 3, ***p ≤ 0.001).

Previous studies have reported that cumulative stress can open the mitochondrial permeability transition pore, consequently leading to mitochondrial membrane depolarization[8]. Mitochondrial membrane potential (ΔΨm) is detected through the measurement of TMRM, a cell-permeant dye with a strong fluorescent signal that accumulates in healthy mitochondria, via flow cytometry. Depolarization of the mitochondrial membrane results in the release of TMRM by the mitochondria, and the fluorescent signal becomes diminished [4]. In addition, we further examined whether EAG affects mitochondrial membrane depolarization. Under EAG treatment conditions, microscopic data showed that fluorescent intensity dramatically decreased (Fig. 6A). Furthermore, membrane potential depolarization was detected after 48 h in EAG-treated Jurkat cells (Figs. 6B and 6C). Collectively, the results show that EAG induced mitochondrial depolarization in leukemia cells.

### Effects of EAG on Reactive Oxygen Species in Jurkat Cells

**Fig. 7:**
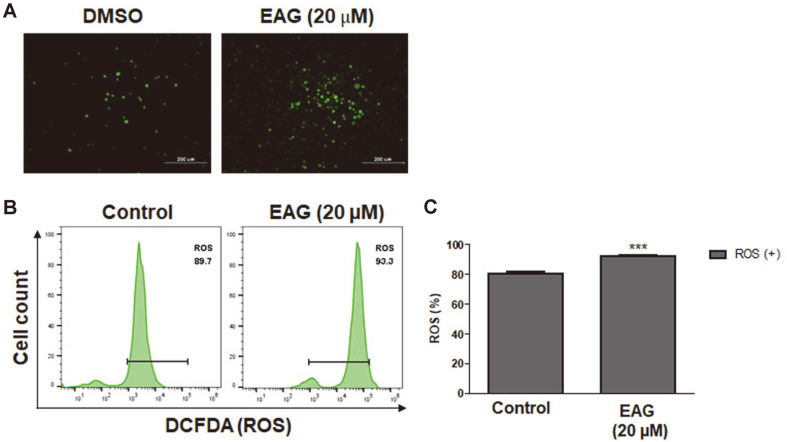
Induction of reactive oxygen species (ROS) levels in Jurkat cells. (**A**) ROS levels visualized through fluorescence microscopy. (**B**) Measurement of ROS levels via flow cytometry with DCFDA. (**C**) Quantification of reactive oxygen species (ROS) levels. Values are represented as means ± SEM. (*n* = 3, ***p ≤ 0.001).

Redox balance of intracellular ROS levels is required for tumor development and progression [18, 19]. ROS can potentially play an essential role in killing cancer cells via elevated oxidative stress through various signaling pathways. Moreover, increased levels of ROS production induced by anti-cancer drugs have been associated with the boosting of apoptosis and cell cycle arrest [21, 30]. To determine whether EAG induces changes in cellular ROS production, the DCFDA ROS detection assay kit was used for ROS level measurement. Confocal microscopy revealed that EAG enhanced ROS in Jurkat cells after 48 h (Fig. 7A). The ROS levels quantified via flow cytometry showed that treatment with EAG increased ROS generation (Figs. 7B and 7C). Thus, these results indicate that EAG-induced ROS production is a potent ROS-induced apoptosis mechanism in Jurkat cells. As such, EAG could potentially serve as a chemotherapeutic agent for leukemic blood cancers.

### Molecular mechanisms of EAG-Treated Jurkat Cells

**Fig. 8:**
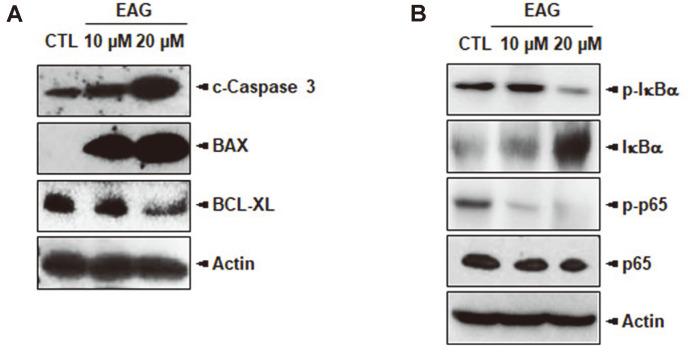
EAG affects apoptosis and NF-κB markers in Jurkat cells. (**A**)The protein expressions of cleaved Caspase 3, BAX, and BCL-XL in the Jurkat cells were examined by western blot analysis. (**B**)The cells were treated with EAG for 48 h before the whole-cell lysates were visualized for p-IκBα, IκBα, p-p65, and p65 expression levels using western blots.

To investigate the specific molecular mechanisms, apoptosis-involved protein levels were analyzed using immunoblots. Our results showed that the expressions of proapoptotic proteins, such as cleaved Caspase 3 and BAX, were upregulated, whereas the expression levels of anti-apoptotic proteins such as BCL-XL were downregulated (Fig. 8A). The NF-κB signaling pathway is mainly involved in cell survival and the proliferation of blood cancers, such as leukemia and lymphoma. Furthermore, p65, also known as RelA, is a key transcription factor that controls several gene expressions associated with oncogenesis. To confirm the anti-proliferation mechanism of EAG, we measured the expression levels of proteins associated with the NF-κB pathway. The results showed that the expressions of phospho-IκBα (p-IκBα) and phospho-p65 (p-p65) were substantially downregulated in Jurkat cells due to EAG treatment (Fig. 8B). Altogether, we observed that EAG promoted cell apoptosis by upregulating pro-apoptotic proteins and inhibiting NF-κB activity.

## Discussion

Leukemia has been reported as a leading form of cancer and a prominent cause of cancer-related deaths worldwide [3]. Recently, the National Cancer Institute estimated that approximately 62,000 people are expected to receive diagnoses of leukemia and predicted that leukemia will cause over 22,000 deaths [20]. The primary treatment for leukemia involves high doses of chemotherapy, and several other methods of treatment have been developed by combining targeted radiation and immune therapy in addition to stem cell transplantation. The earlier a patient is treated, the higher the chance of achieving remission. Historically, plants have been important sources for drug discovery involving natural products, and plant-derived agents such as etoposide, paclitaxel, docetaxel, and irinotecan are among the most effective cancer chemotherapeutic drugs in the anticancer field [5, 6].

Among naturally occurring phenolic substances, coumarins are a large class consisting of fused benzene and α- pyrone rings [1]. These substances are widespread in the plant kingdom, with researchers having discovered over 1,300 types of coumarins as secondary metabolites in a wide array of plants, bacteria, and fungi [7]. In terms of plant biochemistry and physiology, coumarins serve key roles by acting as enzyme inhibitors, antioxidants, and precursors of toxic substances [27]. Moreover, the discovery of diverse pharmacological properties of coumarins in recent years has garnered significant interest, with reports on anti-inflammatory, antioxidant, anti-allergic, hepatoprotective, antithrombotic, antiviral, antituberculosis, anticancer, and other biological activities [27]. Recently, many coumarin derivatives have been isolated from a multitude of plant sources, and the corresponding extracts have been adopted in traditional medicine [15].

The genus *Fraxinus* is one of the 24 genera in the Oleaceae family and comprises 60 species that are widely distributed across temperate environments and subtropical areas in the Northern Hemisphere [28]. Several *Fraxinus* species have been reported to possess medicinal properties, attracting significant interest. Many folk remedies around the world involve the use of such *Fraxinus* species for the treatment of various health concerns, such as constipation, dropsy, arthritis, rheumatic pain, cystitis, and scalp itching [14]. Such species have the trait of containing coumarins, secoiridoids, lignans, flavonoids, and phenylethanoids; most notably, the presence of coumarins distinguishes the genus *Fraxinus* from the other genera of the Oleaceae family [14]. *Flaxinus* coumarins appear mainly in free or glycoside forms, and the coumarin glycosides esculin and fraxin are found in almost all *Fraxinus* species [14].

In a previous study, we reported the isolation of eight coumarins, including two new compounds, from the stem bark of *F. rhynchophylla* and that these coumarins were capable of considerably inhibiting HNE in vitro [17]. Among the isolated coumarins, the coumarin glycoside esculetin 6-O-β-D-arabinofuranosyl-(1→6)-β-D- glucopyranoside (EAG) exhibited significant anti-proliferative activity against Jurkat leukemic cells. We studied the anti-leukemic mechanisms of EAG by investigating the apoptosis-inducing effect of EAG on Jurkat cells in vitro. As a result, treatment with EAG increased early and late apoptotic cells (Fig. 4), leading to the subG0/G1 phase and the S phase giving rise to cell cycle arrest and a decrease in the number of cells, respectively (Fig. 5). In addition, EAG reduced mitochondrial membrane potential in Jurkat cells (Fig. 6). Based on these findings, it can be suggested that intracellular signaling mediated the cytotoxic effect of EAG, which may be based on a mechanism involving mitochondrial ROS upregulation (Fig. 7). In this study, we also demonstrated that the expression levels of the key pro-apoptotic proteins cleaved Caspase 3 and BAX were dramatically increased, whereas that of the anti-apoptotic protein BCL-XL was reduced (Fig. 8A). This may explain the induction of apoptosis after EAG treatment. We then conducted investigations to determine the anti-proliferation mechanism of EAG. NF-κB activation has been observed in several human blood cancers and plays a crucial role in oncogenesis and tumor growth. The NF-κB pathway is stimulated by the IκB kinase (IKK) complex and promotes IκBα phosphorylation, which leads to IκBα degradation. In addition, the pathway allows the p50/p65 heterodimer to be translocated to the nucleus, activating the downstream NF-κB signaling pathway. Our results also demonstrated that EAG induced apoptosis by downregulating p-IκBα and p-p65 in Jurkat cells (Fig. 8B). The experimental results obtained through this research further highlight the potential of EAG as a multi-target therapeutic agent for leukemia.
